# Hysterosalpingography and Endocervical Microbial Assessment of Infertile Women in Ogun State, Nigeria

**DOI:** 10.4314/ejhs.v34i4.3

**Published:** 2024-07

**Authors:** Abiola O Adekoya, Adeniyi K Akiseku, Deboral A Osisanwo, Austin C Egwuogu, Damilola O Egbetayo, Ayodeji A Olatunji

**Affiliations:** 1 Department of Radiology, Olabisi Onabanjo University Teaching Hospital, Sagamu, Nigeria; 2 Department of Obstetrics and Gynaecology, Olabisi Onabanjo University Teaching Hospital, Sagamu, Nigeria; 3 Department of Medical Microbiology and Parasitology, Olabisi Onabanjo University Teaching Hospital, Sagamu

**Keywords:** Genital microbes, Hysterosalpingography, Infertility, Tubal pathology, Uterine pathology

## Abstract

**Background:**

Infertility is a global health issue with varying etiology. This study determined the prevalence and pattern of tubal and uterine pathologies with genital tract microbial infection among infertile women in Ogun State, Nigeria.

**Methods:**

A cross-sectional study was conducted in a year among 230 infertile women aged 20 years and above scheduled for hysterosalpingography (HSG). Samples for high vaginal and endocervical swabs were analyzed as part of the study.

**Results:**

The mean age was 34.65 (6.18) years, and the age group 30 – 39 years had the highest frequency of infertility. The mean duration of infertility was 4.93 (3.88) years, and secondary infertility (77.8%) was higher than primary infertility (22.2%). Tubal pathology was the most common (36.1%), followed by uterine pathology (30.0%), where the tubal blockage was 82.5% and hydrosalpinx was 17.5%. There were 57 (54.8%) and 47 (45.2%) patients with single and bilateral tubal blockage, respectively. Hydrosalpinx was observed in four (20%), nine (45%), and seven patients (35%) with a right, left and bilateral distribution, respectively. Univariate regression analysis showed older women with tubal pathology were 2.01 times more likely to be infertile than the younger patients (95% CI: 1.042 – 4.100, p = 0.005), and patients with longer duration of infertility were 1.1 more likely to be infertile than patients with shorter infertility duration (95% CI: 0.995 – 1.187, p = 0.010). Of the microbes, 33.9% and 22.2% were isolated in the high vaginal and endocervical swabs of participants with tubal pathologies.

**Conclusion:**

Increasing age, infertility duration, and genital microbes are significant risk factors for tubal infertility; hence, their prompt evaluation is essential.

## Introduction

Infertility is defined as the inability of a couple to achieve pregnancy after 12 months or more of regular unprotected sexual intercourse (1). It is a global concern, affecting around 48 million couples and 186 million individuals (2,3). The prevalence of infertility varies across regions, with higher rates observed in Eastern Europe, North Africa/Middle East, Oceania, and Sub-Saharan Africa (4). Infertility rates are approximately 6% and 10% in the United Kingdom and the United States, respectively (5). Sub-Saharan Africa experiences a prevalence ranging from 10.4% in Sudan to 14.8% in Southwestern Nigeria (6,7). It's noteworthy that societal emphasis on female infertility often overshadows its male counterpart.

In the female reproductive system, infertility may be caused by a range of abnormalities of the ovaries, uterus, fallopian tubes, and the endocrine system, among others. Infertility can be primary or secondary (8). Primary infertility is when a person has never achieved a pregnancy, and secondary infertility is when at least one prior pregnancy has been achieved (8). Tubal and uterine pathologies are risk factors for infertility, and tubal pathologies reportedly constitute 35–40% of infertility causes, and this may be due to infections or surgical damage, leading to tubal blockage (9). Genital infections, particularly from Chlamydia trachomatis, Neisseria gonorrhea, and syphilis, significantly contribute to pelvic inflammatory disease (PID) and subsequent tubal disease. In Nigeria, the prevalence of Chlamydia trachomatis and Gonorrhea infections ranges from 17.6–54.7% and 2–2.4%, respectively (4,10-12). Additionally, peritoneal factors like inflammation, infection, tuberculosis, previous surgeries, endometriosis, and ectopic pregnancy can lead to tubal subfertility (13).

Hysterosalpingography (HSG) is an important radiographic procedure for evaluating the fallopian tube, uterus, cervix morphology, and peritoneal cavity status. Despite being an ionizing and invasive procedure, HSG is a readily available and cost-effective alternative to laparoscopy, especially in low-resource environments. It has a sensitivity of 94% and a specificity of 92% in detecting tubal blockage and may offer a therapeutic advantage through tubal flushing and the treatment of proximal obstruction (14.15). A pre-HSG microbial assessment may aid in diagnosing and possibly treating women with genital infections, which may contribute to tubal factor infertility. This study aimed to describe the pre-HSG genital microbial findings and their relationship to hysterosalpingographic findings among women with infertility. It also analyses the impact of structural alterations in HSG on infertility outcomes.

## Materials and Methods

**Study location, design, and participant eligibility**: This prospective cross-sectional study was conducted at Olabisi Onabanjo University Teaching Hospital (OOUTH), Sagamu, Ogun State, in Southwest Nigeria, between October 2021 and September 2022. Two hundred and thirty women aged 20 years and above seeking treatment for infertility at the Gynaecology Out-patient Clinic of our hospital were enrolled using a non-probability simple random sampling method. Hysterosalpingography was performed at the fluoroscopic unit of the hospital's Department of Radiology. Simultaneously, the endocervical and high vaginal samples were also analyzed at the hospital's Department of Microbiology. Written informed consent was obtained from all participants. Exclusion criteria encompassed patients who declined permission, those with a known history of contrast allergy, active pelvic infection, abnormal uterine or vaginal bleeding, and individuals who had undergone recent pelvic surgery or laparotomy.

**Sample size calculation**: The minimum sample size required for the study was calculated using the formula for estimating the proportions of the population for cross-sectional qualitative studies, which is N = Z^2^ p (1-p) divided by d^2^ (16), where the prevalence of infertility in Southwestern Nigeria is reportedly 14.8% (7). Using a prevalence rate of 32%, 95% CI, and 5% precision, N = (1.96)^2^ x 0.15 x 0.85 divided by 0.05^2^ = 193.76 ≈ 194. The attrition rate was 15% of the calculated sample size, making 223.1. To broaden the study base, the figure was rounded up to 230.

**Methodology**: We employed pre-tested and validated interviewer-administered structured questionnaires to gather information. The questionnaire covered socio-demographic details, parity, type and duration of infertility, age at menarche, age at coitarche, induced abortion, contraceptive use, lifetime number of sexual partners, vaginal discharge, and its color.

**Anthropometry**: Body weight in kilogram (kg) was assessed using a standing weighing scale (Seca® 755, Hamburg, Germany) on an even, horizontal hard surface. Height in meters (m) was measured using a stadiometer (Seca® 755, Hamburg, Germany) with the head in the Frankfort plane (17). The body mass index (BMI) in kg/m^2^ was classified as underweight (BMI <17.8), average (BMI = 17.8–25.6), overweight (BMI = 25.7 – 28.7), and obese (BMI > 28.8) (18).

**Patient preparation and sample collection**: Ethical clearance was obtained before the study, and informed consent was taken from participants. Hysterosalpingography (HSG) was scheduled for proliferative phases of the menstrual cycle (7th–12th day) to ensure endometrial thinning, aiding image interpretation, and excluding pregnancy, an absolute contraindication for HSG. Participants with irregular menstrual cycles underwent pregnancy tests. Preceding the procedure, all participants received premedication with 10mg of oral Hyoscine, N-butyl bromide, and 50 mg of Diclofenac approximately 30 minutes before. This premedication aimed to reduce tubal spasms and alleviate post-procedure pelvic pain. Emergency drugs and equipment were available in the fluoroscopic room. Participants changed into sterile gowns and were initially positioned supine for a pelvic scout radiograph in the anteroposterior view.

The lithotomy position was adopted, and the attending radiologist took necessary protective measures. Endocervical and high vaginal swabs were collected before HSG cannulation. A sterile speculum exposed the cervix, and swabs were obtained for microbial assessment. A wet mount of the high vaginal swabs (HVS) was prepared and examined for microbes. Endocervical swabs (ECS) were inoculated on agar and incubated, and Chlamydia trachomatis was assessed using a diagnostic kit (DIAQUICK® Chlamydia cassette of DIALAB® kit).

After swab collection, uterine size and direction were evaluated using a uterine sound. The HSG cannulation involved injecting a water-based contrast medium into the endometrial cavity under fluoroscopic monitoring. Films were taken supine, outlining the uterine cavity and demonstrating contrast spillage into the peritoneal cavity. Participants were informed about potential complications post-procedure, and antibiotics were prescribed as needed. Delayed films were taken 30 minutes after the procedure for better visualization of hydrosalpinx. Two Consultant Radiologists reported HSG images, and findings were recorded.

A Consultant Microbiologist blinded to the participants' radiographic results analyzed all endocervical samples in a laboratory at our hospital. After reading the results, all models were immediately discarded, as there was no ethical approval for storing biological specimens.

The microbial profile and HSG procedure outcome were communicated to participants, and the necessary treatment was provided. Tubal occlusion in the study referred to unilateral or bilateral occlusion or non-visualization of the tube(s) on HSG. All the study participants were followed up at the gynecological clinic, and none developed a post-HSG pelvic infection.

**Data management and statistical analysis**: Data analysis was conducted using Statistical Package for the Social Sciences (SPSS) version 23.0. Continuous variables were expressed as means and standard deviation, while categorical variables were presented as proportions and frequencies. Differences between values were assessed using a student's t-test and ANOVA. Univariate logistic regression evaluated associations between the presence of infertility and the presence of tubal blockage, hydrosalpinx, duration of infertility, presence of microbial infection, and age. The Pearson correlation coefficient (r) was utilized to evaluate relationships between continuous variables, with statistical significance set at p<0.05.

**Ethical consideration**: The study's ethical approval was granted by the Health Research Ethics Committee of the Olabisi Onabanjo University Teaching Hospital (HREC/443/2021).

## Results

**Demographic and clinical characteristics of participants**: A total of 230 female participants, with a mean age of 34.65 (6.18) years (range: 20 – 51 years), were enrolled in the study. Among different age categories, 51 (22.2%) were aged 20–29 years, 128 (55.7%) were aged 30–39 years, 50 (21.7%) were aged 40–49 years, and 1 (0.4%) were aged over 50 years. The mean age at menarche, first sexual intercourse, and menstrual cycle interval were 14.5 (2.08) years, 21.12 (4.25) years, and 27.69 (3.93) days, respectively. The average duration of infertility was 4.93 (3.88) years. Primary and secondary infertility cases represented 51 (22.2%) and 179 (77.8%) of the cohort, respectively, with mean ages of 32.55 (7.37) years and 35.55 (5.68) years, demonstrating a statistically significant difference (p = 0.006). Nulliparity was the most prevalent parity status, accounting for 63.9% of cases. Furthermore, a significant association was found between types of infertility and age groups (p < 0.001). The mean BMI was 28.04 (6.86) kg/m^2^.

**Factors suggesting pelvic inflammatory disease (PID)**: Out of the participants, 120 (52.2%) had a previous medical history (PMH) of abnormal vaginal discharge (AVD). Among those with AVD, the colour distribution was as follows: white (55%), cream (27.5%), brown (8.3%), yellow (8.33%), and pink (0.83%). Approximately 47.8% of participants reported no PMH of AVD. Contraceptive use was observed in 34 (14.8%) participants, with no association detected among age groups (p=0.09). 145 (63%) participants experienced fetal loss and abortion, and 43% reported having multiple sexual partners. Further details regarding the study participants' demographics, obstetrics, and gynecology characteristics can be found in Table 1.

**Table 1:** Demography, obstetric, and gynecologic characteristics of the study participants

Variables	n (%)
Marital status	
Single	11 (4.8)
Married	217 (94.3)
Separated	1 (0.4)
Divorced	1 (0.4)
Tribe	
Yoruba	192 (83.5)
Igbo	37 (16.1)
Hausa	1 (0.4)
Family type	
Monogamy	186 (80.9)
Polygamy	44 (19.1)
Education status	
No formal education	3 (1.3)
Primary	13 (5.7)
Secondary	70 (30.4)
Post-secondary	144 (62.6)
Type of infertility	
Primary	51 (22.2)
Secondary	179 (77.8)
Duration of infertility	
1 – 3	110 (47.8)
4 – 6	65 (28.3)
>6	55 (23.9)
Parity	
0	147 (63.9)
1 – 2	71 (30.9)
>3	12 (5.2)
Number of sexual partners	
1	131 (57.0)
2 – 3	62 (27.0)
4 – 6	33 (14.3)
>6	4 (1.7)
Number of induced abortions	
None	85 (37.0)
1 – 2	118 (51.3)
3 – 4	25 (10.9)
>4	2 (0.9)
Use of contraceptives	
Yes	34 (14.8)
No	196 (85.2)
Recurrent vaginal discharge	
Yes	120 (52.2)
No	110 (47.8)

**Hysterosalpingography findings**: The mean uterine bi-cornual distance was 42.58 (16.96) mm. Uterine characteristics included 10.9% with a bulky pelvic uterus, 19.1% with a bulky uterus extending into the abdomen, and 70% with a normal uterine size. One (0.43%) participant had a congenital uterine shape abnormality. Regular uterine walls were observed in 59.6%, and irregular walls increased with age and in participants with abortion and fetal loss (60.2%). A significant association was found between uterine wall regularity and participants' ages (p = 0.001). Abnormal uterine lucency was seen in 13% of the study participants, and all had secondary infertility, with a higher frequency (63.3%) in the age group 30-39 years and in participants with an abortion history (83.3%) than those without (16.7%). Most participants (76.7%) had normal cervical canal caliber, while 6.2% and 17.1% had bulky and narrowed cervical canals, respectively. Regular and irregular cervical walls were observed in 91.7% and 8.3%, respectively.

**Figure 1:**
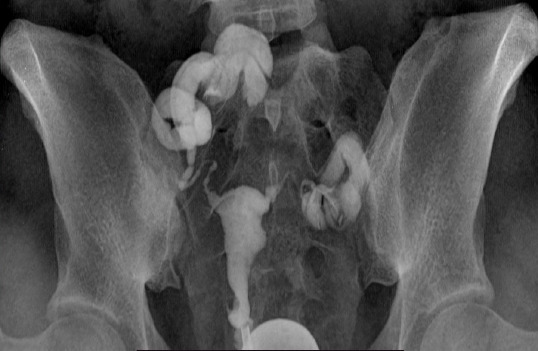
HSG showing a normal uterine cavity with an irregular outline and bilateral hydrosalpinges

Tubal patency and occlusion were noted in 63.9% and 36.1% of participants. Bilateral tubal patency was more common than unilateral tubal patency (72.3% vs 27.7%) (Figure 1). It was higher in secondary infertility cases than in primary cases, with no statistical association (p=0.346). In this study, tubal blockage and hydrosalpinx constitute tubal occlusion and had a distribution frequency of 82.5% and 17.5%, respectively. Tubal blockage was higher in participants with a PMH of abortion (68.3%) than those without (31.7%), but no significant association was observed (p > 0.05). The odds ratio for having a tubal pathology with age and duration of infertility was 2.067 and 1.087, with 95% confidence intervals of 1.042–4.100 and 0.995–1.187, respectively, with statistically significant associations between tubal pathology and age (p = 0.005) and duration of infertility (p = 0.01). However, no associations (p>0.05) were observed with infertility type, BMI, and gynecological characteristics. The tubal characteristics of the study participants are shown in Tables 2 and 3.

**Table 2:** Hysterosalpingography tubal characteristics of the study population

VARIABLES	FREQUENCY (%)
Patent	220 (63.9)
Occluded	124 (36.1)
Patent tubes	
Single left	31 (14.1)
Single right	30 (13.6)
Bilateral patent	159 (72.3)
Occluded tubes	
Hydrosalpinx	20 (16.1)
Blocked	104 (83.9)
Hydrosalpinx	
Right	4 (20)
Left	9 (45)
Bilateral	7 (35)
Blocked	
Single	57 (54.8)
Bilateral	47 (45.2)
Peritoneal loculation	(4.35)

**Table 3:** Relationship between tubal pathology and select participants' variables

Variables	Absent n (%)	Present n (%)	P value
Age (years)			
20-29	32(31.7)	19(14.7)	0.005*
30-39	53(52.5)	75(58.1)	
40-49	15(14.9)	35(27.1)	
>50	1(1)	0(0)	
Number of abortions			
None	38(37.6)	47(36.4)	0.976
1-2	52(51.5)	66(51.2)	
3-4	10(9.9)	15(11.6)	
>4	1(1)	1(0.8)	
History of contraceptive use			
Yes	17(16.8)	17(13.2)	0.439
No	84(83.2)	112(86.8)	
The mean duration of infertility	4.20(3.15)	5.51(4.27)	0.010*
Previous pelvic infection			
Present	53(52.5)	67(51.9)	0.935
Absent	48(47.5)	62(48.1)	
ECS			
Positive	37(36.6)	41(31.8)	0.441
Negative	64(63.4)	88(68.2)	
HVS			
Positive	26(25.7)	25(19.4)	0.249
Negative	75(74.3)	104(80.6)	

ECS – endocervical swab, HVS – high vaginal swab

**Figure 2:**
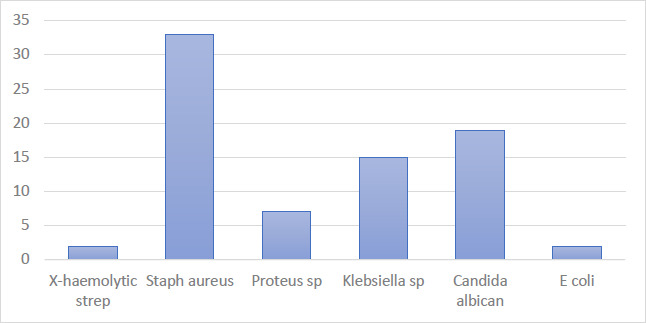
Distribution of the microbial isolates on the high vaginal swab

**Microbial studies**: Genital microorganisms were isolated in 33.9% of wet mount high vaginal swabs (HVS) and 22.2% of endocervical swabs (ECS). Staphylococcus aureus was the predominant organism in HVS, accounting for 42.3% of isolates. Other organisms identified in HVS included Gram-negative bacilli (7.8%), Gram-positive bacilli (6.1%), Candida albicans (4.8%), and Staphylococcus aureus (2.2%). In ECS, Escherichia coli (E. coli), Proteus, and Haemolytic streptococcus were each observed in 0.4% of isolates. (Figure 2) Chlamydia sp. growth was not detected.

The distribution of HVS isolates among participants with tubal pathology was 39.7% for tubal blockage, 29.5% for bilateral patent tubes, 10.3% for unilateral right patent tubes, 10.3% for unilateral left patent tubes, and 10.3% for hydrosalpinx. Among participants with tubal pathology, HVS and ECS isolates were present in 19.4% and 31.8%, respectively. However, no important significance was observed between tubal pathology and isolates (p > 0.05), as shown in Table 4.

**Table 4:** Relationship between tubal pathology and select participants' variables

Variables	Absent n (%)	Present n (%)	P value
**Age (years)**			
20-29	32(31.7)	19(14.7)	0.005*
30-39	53(52.5)	75(58.1)	
40-49	15(14.9)	35(27.1)	
>50	1(1)	0(0)	
**Number of abortions**			
None	38(37.6)	47(36.4)	0.976
1-2	52(51.5)	66(51.2)	
3-4	10(9.9)	15(11.6)	
>4	1(1)	1(0.8)	
**History of contraceptive use**			
Yes	17(16.8)	17(13.2)	0.439
No	84(83.2)	112(86.8)	
The mean duration of infertility	4.20(3.15)	5.51(4.27)	0.010*
**Previous pelvic infection**			
Present	53(52.5)	67(51.9)	0.935
Absent	48(47.5)	62(48.1)	
**ECS**			
Positive	37(36.6)	41(31.8)	0.441
Negative	64(63.4)	88(68.2)	
**HVS**			
Positive	26(25.7)	25(19.4)	0.249
Negative	75(74.3)	104(80.6)	

ECS – endocervical swab, HVS – high vaginal swab

**HSG findings association with demographics, clinical, and microbial data**: A statistically significant association was observed between uterine wall outline and age groups (p = 0.001). An important difference was noted between the presence of tubal pathology and the mean duration of fertility (p=0.01). No significant association was observed between HVS and ECS microbial isolates and any tubal pathology found on HSG (Table 4).

## Discussion

High rates of infertility are a significant issue in Sub-Saharan Africa, particularly in Nigeria, imposing a substantial burden on affected individuals (6,7). The causes of infertility vary based on demographics and population characteristics, underscoring the need for comprehensive studies and appropriate management (19). Unfortunately, societal misconceptions in Sub-Saharan Africa persist, often blaming women for infertility, leading to negative consequences such as divorce, abandonment, and damaged self-esteem (20).

The study primarily focused on women in their reproductive years, indicating early marriage practices. However, the delayed presentation at infertility clinics in their 30s suggests persistent unsuccessful attempts to conceive, emphasizing the urgency for medical intervention. The study noted a prevalent incidence of secondary infertility, aligning with global trends (9,20-22). Unsafe abortions and poorly managed abortions have reportedly increased the incidence of tubal and pelvic infection amongst the affected individuals, resulting in tubal and pelvic infections, which adversely results in a higher incidence of secondary infertility (23).

Tubal pathology, comprising tubal blockage and hydrosalpinx, was the most common finding in this study, with a prevalence comparable to that of reported studies worldwide (9,21,23-25). However, higher incidences were reported in Nigeria and Pakistan (26,27). The high incidence of tubal blockage due to hydrosalpinx was also reported in Nigeria (26), Saudi (9), and India (19). The high prevalence, poor treatment, and recurrence of pelvic infections in our environment may contribute to the prevalence of tubal abnormalities in our study population.

The present study observed hydrosalpinx in 17.5% of cases, a finding higher than most reported studies (20,21). Surprisingly, in our research, unilateral hydrosalpinx was more common than bilateral cases, as previously reported, and contrary to some reports, possibly influenced by geographical variations and the study's prospective nature (9,27). Hydrosalpinx was more on the left than on the right or in bilateral cases, as Christian et al. (26) and Onwuchekwa et al. (28) reported. However, this finding is in dis-concordance with reported cases of bilateral cases or higher incidences of right-sided hydrosalpinx due to the presence of the right-sided location of the appendix, previous appendicitis, or post-surgical complication of appendectomy (20,21,26) and this study is a prospective study. Other findings, such as peritubal adhesion, were reported similarly in the Nigerian study (28). Tubal abnormalities were significantly linked to age and duration of infertility, highlighting the impact of age-related tubal permeability decline and unsafe abortion practices (23).

Uterine anomalies, such as uterine bulkiness, were this study's 2nd commonest HSG finding, as reported by other studies (26,28). However, a survey conducted in Calabar, Nigeria, reported uterine myoma as the commonest HSG finding, with differences attributed to their diet, such as red meat, alcohol intake, and other cultural differences (29). Bulky uterus, from uterine fibroid, is the commonest benign uterine mass in black women of reproductive age (26). Uterine and cervical wall irregularities were the next common uterine abnormality observed in this study, a finding attributed to excessive uterine curettage and infectious morbidities. Only one case of uterine congenital anomaly was reported in our study, a finding similar to another study (23) but lower than those reported by Bukar et al. (30) and Christian et al. (26).

Sexually transmitted infections are common causes of female infertility worldwide, with Chlamydia trachomatis being its leading cause (31). Despite the global prevalence of sexually transmitted infections causing infertility, the absence of Chlamydia trachomatis in this study raised questions. The easy accessibility and frequent use of antibiotics in urban settings may explain this discrepancy (20,32). However, other microbial isolations in participants with tubal pathology suggest a possible association between tubal patency and various microbes beyond Chlamydia and Gonorrhea. In addition, the high prevalence of Chlamydia trachomatis infections was also reported among women with high-risk sexual behavior living in rural areas, aside from infertility (32).

This study has its limitations. The absence of Chlamydia trachomatis isolation may also be associated with the employment of the rapid antigen test, which only detects the presence of current infection rather than the serology test. Other factors and causative agents for tubal blockage, such as endometriosis and genital infections from Neisseria gonorrhea, Mycoplasma hominis, and Mycobacterium tuberculosis, were not accounted for in this present study.

In conclusion, increasing age, infertility duration, and genital microbes are significant risk factors for tubal infertility; hence, their prompt evaluation is essential. Delayed presentation at infertility clinics emphasizes the need for early medical intervention. Tubal pathology, including tubal blockage and hydrosalpinx, was prevalent, possibly due to factors such as unsafe abortions and pelvic infections. Uterine anomalies, particularly uterine bulkiness, were also common findings.

A proactive approach may help tailor treatment strategies and improve patient outcomes by addressing existing infections or microbial imbalances before undergoing HSG procedures. Therefore, recommending a pre-HSG microbial study could be a prudent step toward enhancing infertility's diagnostic accuracy and management in Sub-Saharan Africa, particularly Nigeria.

Early counseling and increased awareness of assisted reproductive technologies are essential for affected couples. These technologies should be accessible and affordable to enhance the chances of successful conception for affected couples. Moreover, adoption should be considered and made accessible for those who do not achieve success with other treatments. Overall, the study emphasizes the need for comprehensive care and support for couples facing infertility challenges in Nigeria.
